# The Pharmacogenomics of Cisplatin-Induced Ototoxicity

**DOI:** 10.3390/life16101599

**Published:** 2026-09-24

**Authors:** David Harman, Chiao Xin Lim, Ganessan Kichenadasse, Janna Morrison, Vijayaprakash Suppiah

**Affiliations:** 1 School of Pharmacy and Biomedical Science, College of Health, Adelaide University, Adelaide, SA 5000, Australia; david.harman@adelaide.edu.au (D.H.); janna.morrison@adelaide.edu.au (J.M.); 2 Discipline of Pharmacy, School of Health and Biomedical Sciences, RMIT University, Bundoora, VIC 3083, Australia; chiao.xin.lim@rmit.edu.au; 3 College of Medicine and Public Health, Flinders Health and Medical Research Institute, Flinders University, Bedford Park, SA 5042, Australia; ganessan.kichenadasse@flinders.edu.au; 4 Department of Medical Oncology, Flinders Medical Centre, Bedford Park, SA 5042, Australia

**Keywords:** cisplatin, ototoxicity, pharmacogenomics

## Abstract

Cisplatin is a first line treatment for a wide range of cancers; one commonly observed adverse drug reaction (ADR) of cisplatin is ototoxicity. Onset of ototoxicity from cisplatin is inconsistent and no single risk factor can reliably predict whether a patient will develop ototoxicity. A genetic factor has been explored for these reasons. This study reviews and summarizes the strongest evidence toward a genetic association. A search of the literature was conducted across three databases for studies exploring the genetic association with cisplatin ototoxicity. 15 unique genes were observed by at least two unique studies with single nucleotide polymorphisms (SNPs), which significantly affected the likelihood of cisplatin-induced ototoxicity. There are many SNPs across a variety of genes that can significantly affect the likelihood of cisplatin-induced ototoxicity. Comparing the findings of studies is difficult because of significant heterogeneity in patient characteristics, treatment plans, and the classification of ototoxicity. Despite this, variants in *ACYP2* (rs1872328), *SLC22A2* (rs316019), and *TLR4* (rs10759932) emerged as the most reproducible findings in this review.

## 1. Introduction

Cisplatin is a cornerstone of treatment for a broad range of solid malignancies in both adult and paediatric populations [1]. Despite its efficacy, cisplatin is associated with a high burden of treatment-limiting toxicities, of which ototoxicity is among the most debilitating and irreversible [2]. The Canadian Pharmacogenomics Network for Drug Safety reported that ototoxicity was, overall, the fifth most frequently occurring adverse drug reaction (ADR) in their database [3]. In the Therapeutic Goods Administration’s (TGA) database of adverse event notifications, deafness is listed as the fifth most common ADR for cisplatin after disease progression, nausea, vomiting, and drug ineffective [1].

Cisplatin-induced ototoxicity typically presents as bilateral, progressive sensorineural hearing loss, initially affecting high frequencies before advancing to frequencies critical for speech perception [4]. This toxicity has substantial long-term consequences for quality of life, particularly in children, where hearing loss may adversely affect language development, academic attainment, and social integration [5].

It has been estimated that 43.2% of patients receiving platinum-based chemotherapy experience hearing loss, with considerable interindividual variability in both susceptibility and severity [6]. Cisplatin ototoxicity is mediated by damage to cochlear outer hair cells, spiral ganglion neurons, and the stria vascularis, resulting in permanent auditory dysfunction [7]. Although several clinical risk factors have been proposed, including cumulative cisplatin dose, age at treatment, radiotherapy exposure, sex, and concurrent ototoxic medications, no single factor reliably predicts which patients will experience clinically significant hearing loss [7,8].

The marked interindividual variability in ototoxic outcomes, even among patients receiving similar treatment regimens, has prompted growing interest in genetic susceptibility. Over the years, several studies have explored associations between single nucleotide polymorphisms (SNPs) and cisplatin-induced ototoxicity, identifying variants in genes involved in drug transport, detoxification, oxidative stress response, and DNA damage repair. However, the translation of these pharmacogenomic findings into clinical practice has been limited by inconsistent replication, small cohort sizes, heterogeneous outcome definitions, and population specific effects. Additionally, patients receiving chemotherapy will often have a nuanced treatment plan, introducing variables such as radiation, comedications, and adjustments during treatment, all of which can be confounding in studying how a single characteristic might impact the likelihood of drug-induced ototoxicity.

Greater clarity regarding which genetic associations are robust and reproducible is essential for the development of clinically meaningful predictive models. Hence, the aim of this literature review was to systematically summarise and critically evaluate genetic polymorphisms that have been associated with cisplatin-induced ototoxicity.

## 2. Materials and Methods

The literature search was conducted in three databases: PubMed, Scopus, and Web of Science, using MeSH and free-text terms. The Boolean operators “AND” and “OR” were used to identify relevant papers with the search terms “(cisplatin OR platinum) AND (ototoxicity OR “hearing loss”) AND (genetic OR pharmacogenomic)”, which were original research articles published up to 22 May 2026. The search was limited to papers available in the English language with data from human subjects.

The title and abstract of each article were reviewed independently by two reviewers, with an independent third reviewer resolving conflicts. Studies were eligible if they (i) included human subjects who had received cisplatin chemotherapy and undergone audiometric testing, (ii) compared the presence of an SNP, and (iii) assessed treatment outcomes in terms of relative hearing loss. Studies focused on animal and cell models were excluded as were studies that had no audiometric data. Studies without genotyping were excluded. Abstracts without full text, posters, study protocols, and review articles were excluded.

The findings of this review are categorised by gene and the specific SNP monitored in the included studies.

## 3. Results

### 3.1. Search Results

The search terms returned 343 results from PubMed, 169 results from Scopus, and 119 results from Web of Science, resulting in a total of 450 unique studies after removal of 181 duplicates. Of these 450 abstracts, 385 were deemed irrelevant to the aim of the review. The full text versions of the 65 remaining studies were then retrieved and assessed for eligibility. Another 18 studies were excluded after full text screening, with 47 original articles included in the final review (Figure 1).

### 3.2. Statistically Significant Associations (Table 1)

To highlight the SNPs that had the strongest evidence for a significant association with cisplatin ototoxicity, this section focuses on SNPs that had at least two unique studies report a statistically significant association. Some genes had multiple SNPs with significant associations; this review focuses on the single SNP with the greatest evidence per gene.

#### 3.2.1. Glutathione-S-Transferases

SNPs in Glutathione S-transferases (*GSTs*) are promising research candidates as they aid in the detoxification and metabolism of cisplatin by conjugating glutathione to cisplatin, restricting cisplatin’s ability to bind to DNA. They also protect cells from the stress caused by free radicals [9]. The investigated SNPs in *GSTP1*, *GSTM3*, and *GSTT1* were associated with decreased enzymatic activity, leading to greater likelihood of ototoxicity due to greater damage from free radicals generated by cisplatin [10].

*Glutathione-S-Transferase Pi-1 (GSTP1)*

*Protective effect*

The G/G genotype of *GSTP1* rs1695 had a statistically significant (*p* < 0.001) protective effect in 173 adult testicular cancer survivors (odds ratio (OR): 0.24) [11]. This study graded hearing loss of participants using the Engdahl criteria which, using audiometric data taken from 23,446 Norwegian males, categorised severity by percentile hearing loss (10th, 25th, 50th, 75th, and 90th) at 4 kHz [12]. This categorisation is unique to this study. The majority of studies in this review used grading criteria based on decibel levels at a range of frequencies.

Using the Brock criteria [13], a French study in 106 paediatric patients treated with cisplatin validated [rs1695 (G) OR = 0.27 (*p* = 0.046)] the findings from study [11]. Notably, patients were categorised into two groups according to their hearing loss: grade ≥2 (*n* = 33), and no hearing loss: grade = 0 (*n* = 73) [14].

*Increased risk*

Several studies have also observed an increased risk of cisplatin-induced ototoxicity in patients carrying the G allele of the *GSTP1* rs1695 SNP.

In a study with 188 male patients treated with cisplatin for germ-cell testicular cancer, the G allele of *GSTP1* rs1695 was significantly associated with an increased risk of ototoxicity (OR = 2.97, *p* = 0.049). This study used a modified version of the Common Terminology Criteria for Adverse Events (CTCAE) Version 4.03, which incorporated elements from the American Speech–Language–Hearing Association (ASHA) guidelines to grade ototoxicity [15].

In another study with 61 paediatric patients receiving platinum agents, the rs1695*G allele was associated with an increased risk of ototoxicity. This study used the International Society of Paediatric Oncology (SIOP) scale, which has elements from the CTCAE, Brock, Chang and Muenster criteria [13]. An increased risk of low-frequency hearing loss was observed for carriers of at least one G allele (*p* = 0.02 OR = 10). This study reported that carriage of this allele was associated with any ototoxicity and the likelihood of more severe ototoxicity [16].

In a more recent study that utilised the Brock criteria, 64 childhood cancer survivors treated with platinum agents were divided into two groups; “no hearing loss”, defined as grade 0 or no impactful change to hearing, and “hearing loss”, defined as grades 2, 3, or 4 with moderate to severe hearing loss. There was a significant (*p* = 0.035) association between carriers of the rs1695*G allele and hearing loss [17].

Another study in 89 Brazilian patients with head and neck squamous cell carcinoma (HNSCC) patients treated with cisplatin and radiotherapy were grouped into grades 0–2 and grades 3–4 according to the CTCAE v4.0 criteria. Carriers of at least one rs1695*G allele had an increased hearing loss OR of 4.3 (*p* = 0.01). The likelihood of ototoxicity greatly increased when carriers of at least one rs1695*G allele also carried the A/A polymorphisms of *XPC* rs2228001 (OR = 32.22, *p* = 0.004 PA-97%), *XPD* rs1799793 (OR = 19.44, *p* = 0.02 PA = 92%), or *EXO1* rs1047840 (OR = 12.08, *p* = 0.01 PA = 92%) [18].

The Brock and the Muenster criteria were both employed in a cohort of 72 Turkish paediatric patients. The Muenster criteria is unique in that it has three subgrades (a, b, and c) for grades 2 and 3, and in a comparative study on grading criteria it detected hearing loss earliest [13]. Both criteria found significant associations between the G/G genotype and ototoxicity (*p* = 0.03 using Brock criteria; *p* = 0.01 using Muenster criteria). However, the statistical significance was lost on correction for multiple testing (*p* = 0.093 Brock; *p* = 0.057 Muenster). The authors noted the large confidence interval in the effect of the SNP of interest was likely due to the small sample size of carriers of the G/G genotype (*n* = 7) [10].

*Glutathione-S-Transferase Theta-1 (GSTT1)*

Using the Brock criteria in a cohort of 68 Thai paediatric patients, the wild genotype of *GSTT1* was associated with 10 times the risk of ototoxicity compared to non-carriers (*p* = 0.023) [19].

In a prospective study with 55 adult patients, audiometric testing was carried out at baseline and before and after each cycle of treatment. The authors defined ototoxicity using a simplified version of the Muenster classification where a decrease in hearing by ≥10 dB in ≥2 frequencies or ≥15 dB in 1 frequency in the audiogram was declared an ototoxic response. Carriers of two copies of *GSTT1* were significantly more likely to have worse hearing outcomes at the audiograms before the second cycle (OR = 6.4), after the second cycle (OR = 3.3), and before the third cycle (OR = 6.3) [20].

The total deletion of *GSTT1* was also investigated in multiple studies. Genetic variations where individuals carried only one or no copies of the gene had differences in the prevalence of ototoxicity than in those who carried two copies of this gene.

Another study found a statistically significant protective effect of the *GSTT1* deletion and ototoxicity (*p* = 0.038, OR 3.53) in a cohort of 106 paediatric patients under the Brock criteria [14].

In a Japanese study involving 26 adult head and neck cancer patients, the null genotype of *GSTT1* was associated with ototoxicity. Ototoxicity was measured using a modified version of the TUNE grading system, and patients were separated into early ototoxicity present or absent groups. The TUNE grading system as followed in this study uses the threshold shifts at 8 kHz to define grades one and two based shifts of ≥10 dB and ≥20 dB, respectively, and the pure tone averages of 1, 2, and 4 kHz to define grades three and four as ≥35 dB and ≥70 dB, respectively. *GSTT1* null carriers had an OR of 8.4 (*p* = 0.04) [21].

In a second study the *GSTT1* null was reported as a strong predictor for bilateral ototoxicity [OR of 4.31 (*p* = 0.033)]. However, this significance was lost when correction was applied to multiple testing [22].

*Glutathione-S-Transferase Mu-1 (GSTM1)*

In 118 adult patients, it was observed that the *GSTM1* deletion was significantly more frequent in patients experiencing hearing loss in only one ear. The patients in the study were separated by unilateral or bilateral damage, and *GSTM1* deletion was reported as a predictor for unilateral ototoxicity with an OR of 4.5. Further research is needed in this area as the reason for this unilateral effect was not fully understood [22].

The Macedo study found that combination of *GSTM1* null carriage with carriage of the *XPC* risk allele had an OR of 8.2 of cisplatin-induced ototoxicity in their study cohort of 89 adult patients [18].

However, a Japanese study reported the opposite effect of the *GSTM1* deletion being protective from ototoxicity [OR of 0.10 (*p* = 0.001)] [21].

#### 3.2.2. Acylphosphatase 2 (ACYP2)

Acylphosphatase 2 encodes for an enzyme that hydrolyses the carboxyl-phosphate bond of acylphosphate and is expressed in the cochlear [23]. The SNP rs1872328 in *ACYP2* has been related to cochlear hair-cell damage [24].

In a genome-wide association study (GWAS) in 238 paediatric patients with brain tumours, the *ACYP2* rs1872328*A allele was associated with ototoxicity [OR of 4.5 (*p* = 3.9 × 10^−8^)], which was replicated in an independent cohort of 68 paediatric patients. The severity of ototoxicity was also associated with this A allele. Of note, patients in both cohorts also received radiotherapy [25]. Since then, this finding has been replicated in several studies involving both paediatric and adult patients, which have shown statistically significant associations between the *ACYP2* rs1872328*A allele and ototoxicity [24,26,27,28].

#### 3.2.3. Xeroderma Pigmentosum Complementation Groups

Xeroderma pigmentosum complementation group C (*XPC*) is part of the nucleotide excision repair pathway. The lesions on DNA caused by cisplatin can be removed as part of this pathway. The SNP rs2228001 decreases the genetic activity of *XPC*, which in turn increases ototoxicity [18,29].

A Spanish study that included both adult and paediatric patients (*n* = 32) showed that C homozygotes of *XPC* rs2228001 were associated with ototoxicity [OR of 17.16 (*p* = 0.042)] [29].

A later study investigated how polymorphisms in the nucleotide excision repair pathway can alter patient outcomes from cisplatin chemotherapy. This study was conducted with 90 patients who had received cisplatin and radiotherapy for HNSCC treatment. CTCAE v4.0 was used to grade ototoxicity, and patients were sorted into groups of grades 0–1 and grades 2–4. Interestingly, they found a statistically significant reduction in the risk of moderate to severe ototoxicity in patients with the A/C and C/C genotypes of *XPC* rs2228001 OR = 0.20 (*p* = 0.01) [30]. This finding was independently replicated by the Macedo study [OR of 3.13 (*p* = 0.01)] [18].

Xeroderma pigmentosum complementation group D (*XPD*) is another part of the nucleotide excision repair pathway and the SNP rs1799793 results in reduced genetic expression much the same as the *XPC* variant [30].

Carriers of *XPD* rs1799793*A/A were reported with an OR of 12.29 (*p* = 0.03). When analysis was performed on carriers of just one A allele, there was no noticeable difference in the likelihood of ototoxicity when compared to G/G carriers [30].

An independent association was not found between carriers of *XPD* rs1799793 and ototoxicity. When analysis was refined to carriers of the rs1799793*A/A genotype who were also carriers of a G allele in *GSTP1* rs1695, a large OR of 19.44 (*p* = 0.02) was observed [18].

#### 3.2.4. Methyltransferases

*Thiopurine methyltransferase (TPMT) and Catechol-O-Methyltransferase (COMT)*

Thiopurine methyltransferase encodes an enzyme which uses S-adenosylmethionine (SAM) as a substrate in the methionine pathway [31]. SAM is a substance that is known to increase the toxicity of cisplatin. Catechol-O-Methyltransferase also relies on SAM as a methyl-donor substrate. A decrease in the activities of TPMT and COMT result in higher levels of SAM and greater ototoxic damage [20,32].

The first study to find an association between variants in *TPMT* and *COMT* and ototoxicity had two cohorts of Canadian paediatric patients (*n* = 53 and 109). The cohorts were genotyped for 1949 unique SNPs across a variety of genes related to drug absorption, distribution, metabolism, and elimination. CTCAE was used to grade hearing loss, with patients sorted into significant hearing-loss absent (grade 0) and present (grades 2–4). The combined cohort had significant associations in three variants in *TPMT* and two in *COMT*. The study also found younger age and male sex to be significant factors that increased the risk of ototoxicity. After regression analysis with correction for age and sex, *TPMT* rs12201199 A allele [OR of 16.98 (*p* = 0.03)] and *COMT* rs9332377 A allele [OR of 5.52 (*p* = 0.03)] were statistically significant [31].

A Canadian study genotyped 155 paediatric patients for previously associated variants in *TPMT* and *COMT*. Using the CTCAE v3 criteria, associations were observed for all three *TPMT* variants: rs12201199*A allele OR = 6.1 (*p* = 0.0013), rs1142345*G allele OR = 4.5 (*p* = 0.011), and rs1800460*A allele OR = 3.6 (*p* = 0.038) [33].

A Canadian study investigated the three previously associated *TPMT* variants in 371 patients [33]. Patients possessing multiple ”risk alleles” were at greater risk than those carrying only one ”risk allele”. The effect on ototoxicity was reported at an OR = 1.41 *p* = 0.038 per risk variant [32].

A 2016 study found a statistically significant increase in the risk of ototoxicity in carriers of the *COMT* rs9332377*T allele at three treatment points: before the second cycle (OR = 5.4), after the second cycle (OR = 3.9), and before the third cycle (OR = 6.5) [20]. In a subsequent study in 206 adult patients with HNSCC graded for ototoxicity by the CTCAE v4.03 criteria, older age and cisplatin dosage were found to increase the risk of ototoxicity. After correction, the *COMT* rs9332377*T allele was found to have an OR = 1.72 (*p* = 0.046) [34].

#### 3.2.5. Superoxide Dismutase 2

The rs4880 variation in the superoxide dismutase 2 gene (*SOD2*) had previously been associated with noise-induced hearing loss [35] and was the rationale for its selection as the candidate gene in a cisplatin ototoxicity study [36]. *SOD2* encodes for manganese superoxide dismutase (*MnSOD*), a component of mitochondrial superoxide anion detoxification. Superoxides are dismutased into hydrogen peroxide by *MnSOD*. The rs4880 variation was found to lead to increased MnSOD activity while cisplatin treatment reduced other mitochondrial antioxidant factors, which metabolise the hydrogen peroxide. The combined effect may have resulted in a build-up of hydrogen peroxide that leads to cellular damage in the outer hair cells of the cochlear [36]. The rs4880 variant may also impact the shape of the enzyme, which was hypothesised to restrict its entry into cells to metabolise ROS, leading to increased cell death [37].

Seventy-one paediatric patients with medulloblastoma were genotyped for five different *SOD2* genetic variants. In this study, hearing loss was simply defined as the need for hearing aids after starting treatment. Covariates that were adjusted for included age at diagnosis, sex, ethnic group, cumulative cisplatin dose, and radiation dose. Following correction, the study reported three times greater risk of ototoxicity with the rs4880*C allele [36]. In a later GWAS study in 393 Danish patients treated with cisplatin for testicular cancer, age and cisplatin dosage significantly impacted ototoxicity; after correction, the *SOD2* rs4880*A allele had an OR of 1.55 *p* = 0.007 [37].

#### 3.2.6. Otoprotective Variants

*Solute carrier family 22 member 2 (SLC22A2)*

Solute carrier family 22 member 2 (*SLC22A2*) encodes the organic cation transporter 2 (*OCT2*) that is involved in the transport of cationic compounds across plasma membranes, which would include the entry of cisplatin into the cochlear. The variant rs316019 was associated with mild cisplatin ototoxicity, implying it may be a hypomorphic variant [38]. The variant of *SLC22A2* has a reduced affinity for cisplatin, which may lead to a decrease in exposure of cisplatin to the cochlear, reducing cellular damage and ototoxicity [39].

A cohort of 64 German paediatric patients was genotyped for SNPs in SLC transporter genes. This study used the Muenster classification system to grade ototoxicity. After correction for age at the start of treatment (found to have an OR of 0.84 per year), the *SLC22A2* rs316019*T allele was observed to have an otoprotective effect (OR = 0.12 *p* = 0.009). This finding was replicated in a second cohort of 66 adult patients [39].

A study in South Africa genotyped a cohort of 214 patients for five candidate genes. Even though this study employed three unique grading criteria for ototoxicity, the ASHA, Chang, and CTCAE criteria, the *SLC22A2* rs316019*T allele was associated with reduced rates of ototoxicity by the Chang criteria [OR = 0.439 (*p* = 0.039)]. Interestingly, while the CTCAE criteria had a non-significant decrease in risk, the ASHA criteria showed a non-significant increased risk of ototoxicity [40].

The PanCareLIFE study from across Europe tried to validate previous genetic findings for cisplatin ototoxicity in 741 patients who had received cisplatin treatment. The Muenster grading criteria were used to assess hearing loss. Of the genetic markers genotyped in the study, only *SLC22A2* rs316019 showed statistical significance. Instead of the T allele, the C allele was the focus of this study. The C allele increased the risk of ototoxicity; OR 1.51 (*p* = 0.042). This was in agreement with other papers that suggested the T allele is protective. Age, cranial irradiation, and cisplatin dosage were reported to have significant impact on hearing loss in this cohort [38].

*Nuclear Factor Erythroid 2-Related Factor 2 (NFE2L2)*

Nuclear Factor Erythroid 2-Related Factor 2 (*NFE2L2*) produces the protein NRF2, which is a transcription factor involved in the protection of cells from oxidative and other stresses. The rs6721961 variant is located in the promoter region of the gene and interferes with a binding site of the protein, inhibiting the positive feedback loop of self-expression regulation and leading to a 40% reduction in expression and increasing the risk of ototoxicity [40].

The first study to utilise *NFE2L2* as a candidate gene found *NFE2L2* rs6721961*A to have significant protection against ototoxicity when hearing loss was graded with ASHA (OR = 0.44 *p* = 0.039) and the CTCAE criteria (OR = 0.42 *p* = 0.034). The third and final criteria used in this study, the Chang criteria, showed a similar trend toward protection, although it did not reach statistical significance (*p* = 0.238) [40].

The Fujikawa study also reported a statistically significant result with the G allele of the *NFE2L2* variant (OR = 9.1 *p* = 0.03). It is worth noting the small number of participants and that every carrier of this risk allele was also a carrier of the *GSTT1* deletion, which the paper also found to have a significant increase in risk [21].

*Multidrug and toxin extrusion protein 1 (MATE1)*

Multidrug and toxin extrusion protein 1 (*MATE1*) rs2289669 variant offers increased protection from ototoxicity, possibly due to enhanced efflux of cisplatin [34]. MATE1 is a bidirectional antiporter that is expressed in the inner ear and likely interacts with OCT2 in the distribution of cisplatin [41].

One study found that carriers of the A/A genotype of *MATE1* rs2289669 had significant protection against ototoxicity [OR 0.46 (*p* = 0.01)] [34].

A retrospective study conducted in the Netherlands aiming to enhance the accuracy of a predictive model for cisplatin-induced hearing loss included 74 patients genotyped for 31 previously associated SNPs. Hearing loss was measured by change in decibels at 1 2–4 kHz post-treatment. The A/A genotype of *SLC471/MATE1* rs2289669 was the only significant predictor of hearing loss, with a 2.67 DB difference (*p* = 0.017) [41].

*Toll-Like Receptor 4 (TLR4)*

Toll-Like Receptor 4 (*TLR4*) expresses a cell surface receptor that is involved in immune responses to stress and pathogens. Animal studies found that inhibition or removal of TLR4 led to reduction in cell death and ototoxicity [42]. Cisplatin has been linked to increased expression of TLR4 and the activation of intracellular proinflammatory signalling in the inner ear. Reduced expression of TLR4 due to variants such as rs10759932 lead to protection from ototoxicity [43].

When comparing the “hearing-loss present” cohort (CTCAE v5 grades 2–4) with the ”hearing-loss absent” cohort (grade 0), *TLR4* rs10759932*C allele carriers had an OR = 0.34 (*p* = 0.0007) [42].

Several genetic variants/SNPs in *TLR4* were investigated in a cohort of 213 adult and 357 paediatric patients from Canada. Cisplatin and radiation dosage in the adult cohort, and age and sex in the paediatric cohort, respectively, were predictors of ototoxicity. In both cohorts, the CTCAE v4.03 criterion was used to grade ototoxicity. The SNPs studied were prioritised based on their combined annotation dependent depletion (CADD) score, a common measure of variant deleteriousness. In the combined cohort, the SNP with the highest CADD score was the *TLR4* rs10759932*C allele, with an OR of 0.34 [43].

#### 3.2.7. Mismatch Repair Pathway-Associated Genes

*Exonuclease 1 (EXO1) and MutS homolog 3 (MSH3)*

Exonuclease 1 (*EXO1*) and MutS homolog 3 (*MSH3*) both play a role in encoding the mismatch repair pathway, which repairs DNA damage from cisplatin. A reduction in genetic activity from the genetic variants in these genes reduces the ability to repair, which increases the damage from cisplatin [18,44].

Two papers from Brazil found associations in SNPs in *MSH3* and *EXO1*. The first study included 90 patients with HNSCC who were treated with cisplatin and radiotherapy and genotyped for the *EXO1* SNPs rs1047840 and rs9350 and *MSH3* SNP rs26279. While neither *EXO1* SNPs were independently associated with ototoxicity, the combination of the rs1047840*T allele (no T/T homozygous patients were found in this cohort) and rs9350*G allele led to a significant increase in ototoxicity [OR 4.00 (*p* = 0.03)]. Carriers of the *MSH3* rs26279*G allele had a significantly increased risk of ototoxicity [OR 10.29 (*p* = 0.04)] [44].

The second paper reported nine significant associations, including combinations of genetic variations in multiple genes. Patients who were *EXO1* rs1047840 A homozygotes who also carried the *GSTP1* rs1695*G allele had a 12.08 times increased risk of severe hearing loss. Combination of the rs1047840*A allele and C/C homozygosity for *P53* rs1042522 had an OR of 20.97. Carriers of the *MSH3* rs26279*G allele and *XPC* rs2228001 A/A had an OR of 17.09 [18,24].

**Table 1:** Summary of significant findings (odds ratios) for genetic association with ototoxicity.

	Gene	*ACYP2*	*SLC22A2*	*TLR4*	*XPD*	*GSTT1*	*SOD2*	*MSH3*	*EXO1*	*XPC*	*GSTP1*	*GSTM1*	*MATE1*	*NFE2L2*	*COMT*	*TPMT*
Study																
[42]				0.36												
[45]						10						x				
[36]							3.06									
[22]						x						x				
[29]										17.2						
[19]						x						x				
[46]		x	x				x				x			x		x
[28]		N/S													x	x
[15]											2.97					
[24]		14.7														
[41]													N/S			
[21]		x	x			8.4					x	0.10		9.1		x
[37]							1.55									
[47]															x	x
[38]		x	1.51				x				x			x		x
[48]															x	x
[39]			0.12													
[43]				0.34												
[16]											N/S					
[30]					12.3					0.2						
[14]						3.53	x			x	0.27	3.53				
[18]					19.4 *	x		17.1 *	12.1 *	3.13	4.3	8.2				
[44]								10.3	4.0 *							
[11]						x					0.24	x				
[10]											x				x	x
[9]						x					x	x				
[33]															x	6.1
[31]															5.4	17
[17]											N/S					
[32]																1.41
[40]			0.44											0.11		
[20]						6.36	x				x				5.45	x
[34]		x											0.46		1.72	x
[27]		5.91													x	x
[26]		12														
[25]		4.5														
[49]															x	x

Genes are ordered from left to right by highest strength of evidence to lowest. * = when carried in combination with another variant allele; grey x = no statistically significant relationship observed; N/S = odds ratio not stated; blue = protective effect; orange = increased risk.

## 4. Discussion

This review included 47 published studies investigating genetic susceptibility to or protection from cisplatin-induced ototoxicity. From these studies, statistically significant associations were found in SNPs across 31 unique genes. Of these 31 genes, only 15 had their statistically significant associations observed in more than one study. Interestingly, 14 studies reported no significant findings. While these studies have highlighted the biological plausibility of genetic contributions to ototoxicity risk, substantial methodological heterogeneity has impeded translation into the clinical setting.

Only a small subset of SNPs showed statistically significant associations across multiple independent cohorts (Table 1). Variants in *ACYP2* (rs1872328), *SLC22A2* (rs316019), and *TLR4* (rs10759932) emerged as the most reproducible findings in this review. Notably, these variants showed consistent directionality of effect across studies and were associated with ototoxicity independently of other genetic modifiers. The *ACYP2* rs1872328 variant, initially identified through GWAS in paediatric patients, has since been replicated in both paediatric and adult cohorts, lending credence to its role in cisplatin-induced cochlear damage. Similarly, the protective effect of the *SLC22A2* rs316019 T allele has been observed across multiple populations, consistent with its proposed role as a hypomorphic variant that reduces cisplatin uptake into cochlear cells. More recently, downregulation of TLR4 signalling mediated by rs10759932 has been associated with reduced ototoxicity risk, supporting a mechanistic role for inflammatory pathways in cisplatin-induced inner ear injury.

In contrast, many SNPs had inconsistent replication across studies. Fifteen SNPs were identified as significant in single studies only, often without functional validation or independent confirmation. These associations, including variants in glutathione S transferase genes, transporters, and genes involved in cisplatin metabolism-related pathways, demonstrated inconsistent replication or population specific effects. These findings underscore the importance of cautious interpretation of isolated associations, particularly when derived from small cohorts or post hoc subgroup analyses.

A major contributor to inconsistent findings across studies is the lack of harmonisation in phenotyping ototoxicity. The gold standard for measuring hearing loss is pure tone audiometry, but there are multiple ways in which results from this testing can be interpreted. In the included studies at least 14 different criteria had been used to define and grade hearing loss, including multiple versions of the NCI CTCAE, Brock, Chang, Muenster, SIOP, ASHA, and institution specific modifications. Two studies did not specify how they interpreted the audiometric readings while eleven studies used multiple criteria when grading hearing loss, such as [40], which used three different grading scales: ASHA, Chang, and CTCAE. In this study, the three grading scales not only varied on the significance of their findings, but in the case of the *SLC22A2* variant, also the direction of the variant’s effect. The study reported in [36] defined hearing loss as the need for hearing aids in patients post-treatment. This criterion could have excluded some milder, yet significant hearing loss. The typical threshold at which hearing aids are considered is a loss >20 dB at 3000 Hz, which is past the threshold for impactful hearing loss on multiple scales, including CTCAE and Muenster [46]. The study reported in [16] found low frequency hearing loss rather than general hearing loss, the relevance being that these lower frequencies are typical of more severe hearing loss and may include the frequencies in speech.

Patient and medication characteristics, such as age, cisplatin and/or radiation dosage, sex, and co-administered medications, vary between studies, as does the impact of each factor on ototoxicity. Age was often only a significant factor in groups of very young or very old patients where the outer hair cells of the cochlea, whose vibration is responsible for hearing, have not fully formed or have already somewhat degraded due to aging [50,51]. Of the 47 studies in this review, 23 found cisplatin dosage to have a significant impact [11,14,15,16,18,21,27,30,32,34,37,38,40,41,42,43,44,46,49,52,53,54,55], 23 studies found the effect insignificant [9,10,17,19,20,22,25,26,28,29,31,33,36,39,45,47,48,56,57,58,59,60,61], and one study did not state whether cisplatin dosage/schedule had a significant impact on ototoxicity [24].

Many studies adjusted for male sex as a covariable in multivariate analysis, but only seven studies found male sex to independently associate with a significant increase in the risk of ototoxicity. While the trend typically shows an increase in risk, the influence of male sex may be too small to be a clinically important predictor of ototoxicity [62,63]. Only two offered an OR for male sex, OR = 1.59 in [32] and OR = 6.2 under the Muenster criteria, or 3.4 under the Brock criteria in [10]. All seven studies that reported the sex association had a paediatric cohort [10,31,32,33,36,43,49]. Although not included in this review, one paper did observe an OR of 2 for male sex and ototoxicity in an adult cohort, although their study was not specific to cisplatin [63].

Radiation therapy is known to increase the risk of ototoxicity and the use of radiotherapy varied between cohorts in the studies. One study found an association for carriers of *TCERG1L* rs893507 and ototoxicity in their cohort of patients who had not received cranial irradiation as part of their therapy [57]. When a cohort of patients who had received cranial irradiation was included in their analysis, the significance of this finding was lost as the influence from radiotherapy was more impactful and potentially overrode any genetic associations [57]. Of the 29 studies that included patients who received radiotherapy, only 12 consisted entirely of patients who had received radiotherapy [11,18,21,25,28,30,34,41,45,47,49,56]. If not accounted for, this confounding factor can overestimate the effects of genetic variants. The remaining studies in this review included five that did not specify if patients had received radiotherapy [37,40,42,52,54] and thirteen that excluded radiotherapy recipients [14,16,22,26,29,39,48,51,53,55,58,60,61].

The variety in cohort size was notable across the studies, ranging from 26 [21] to 1680 participants [55]. Half of the studies were conducted in Europe (*n* = 24), 15 in North America, four each in Asia and South America, and three in Africa. While the diversity in cohort locations, and by extension genetic backgrounds, is beneficial to the robustness of the findings, there is a substantial gap in ethnic diversity, with fewer than five studies in Asia, South America, and Africa.

For a pharmacogenomic association to be implemented in clinical settings, replication of causational relationships in independent cohorts is a necessary yet historically challenging milestone [64]. Of the six GWAS studies included in this review, only three had significant findings [37,42,57]. From these findings, only two SNPs were successfully replicated in independent cohorts. The strength of these newly identified associations can be overestimated in their discovery cohort and struggle to be replicated. This phenomenon referred to as the “winner’s curse,” reduces the likelihood of successful replication, especially when validation cohorts differ in ancestry, treatment protocols, or phenotype definitions [65]. Candidate gene studies, although hypothesis-driven, often suffer from insufficient statistical power and inconsistent correction for multiple testing.

A polygenic approach to ototoxicity prediction shows promise. Few associations had strength where they could be clinically applied. By analysing a cohort carrying multiple genetic variants implicated in ototoxicity, the strength of association will theoretically increase as seen in studies [18,34]. Securing a large enough cohort of carriers for a sufficiently powered polygenic study is a major challenge faced by researchers. Many of the variant allele frequencies (VAFs) for SNPs of interest, especially in *ACYP* and *TPMT*, are small (~5%) making it unlikely that people will possess both SNPs. Compounding this is the great difficulty in compiling the findings from multiple studies due to significant heterogeneity in the studies and results that cannot easily be re-analysed, i.e., audiometric results presented through aberrant grading criteria and studies not declaring their observed MAF/variant count. 

There are limitations of this review regarding both the heterogeneity between studies and patient confidentiality restricting the ability to compile all data gathered across unique studies and the subsequent analysis of all evidence for individual genetic variants as a complete dataset. The quality of the findings of study [31], which was the first to observe a statistically significant association between variants in *TPMT* and *COMT* and cisplatin ototoxicity, have also come into question in recent years [66]. *TPMT* was evaluated as a candidate gene in more studies than any other gene in this review but had the lowest replicability. The study reported in [31] has had flaws pointed out, namely the failure to match covariates like vincristine exposure, which call into question the validity of the original findings [66]. Given the complexity of the field of pharmacogenomics and the limited replicability observed in this review, the genetic variants nominated as having the greatest evidence for cisplatin ototoxicity should be taken as recommendations for avenues of further research before they have clinical implementation.

Progress toward clinical implementation will require coordinated, multicentre efforts with standardised audiometric phenotyping, transparent reporting of variant frequencies, and inclusion of ethnically diverse populations. Prospective studies integrating pharmacogenomic data with cumulative dose exposure and longitudinal hearing assessments are particularly needed. Ultimately, the integration of robust genetic markers into decision support frameworks may enable risk-stratified treatment planning, enhanced audiological surveillance, and targeted otoprotective interventions such as a reducing the administered dose of cisplatin or selection of less-ototoxic medication like carboplatin or nedaplatin.

## Figures and Tables

**Figure 1:**
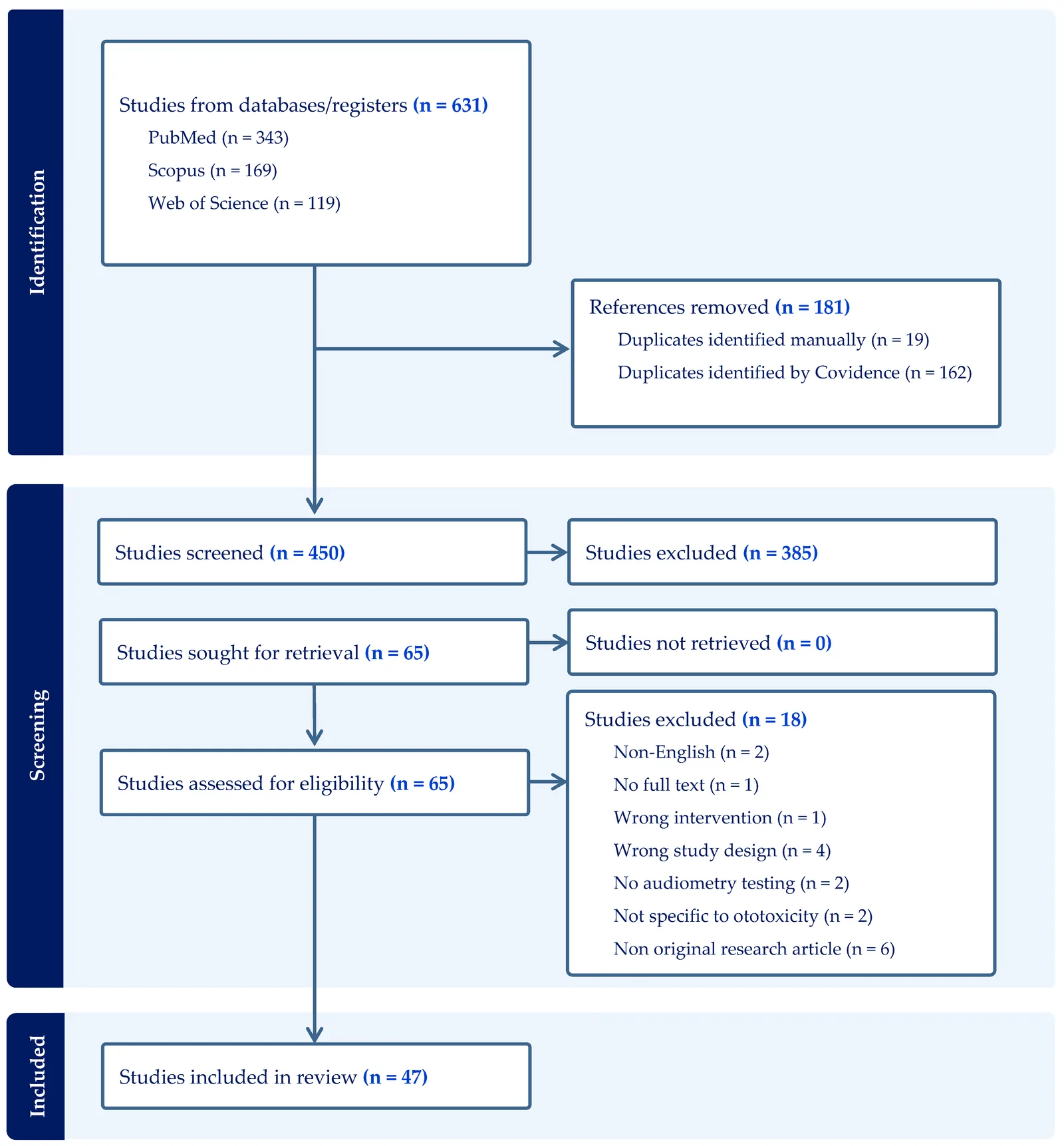
PRISMA flowchart of the literature search.

## Data Availability

The data presented in this study are available on request from the corresponding author.

## Abbreviations

The following abbreviations are used in this manuscript:

ADR	Adverse Drug Reaction
SNP	Single Nucleotide Polymorphism
TGA	Therapeutic Goods Administration
OR	Odds Ratio
CTCAE	Common Terminology Criteria for Adverse Events
ASHA	American Speech–Language–Hearing Association
SIOP	International Society of Paediatric Oncology
HNSCC	Head and Neck Squamous Cell Carcinoma
SAM	S-adenosylmethionine
CADD	Combined Annotation Dependent Depletion
VAF	Variant Allele Frequency
